# Early Functional Outcomes and Complications of the First Carpometacarpal Arthroplasty: A Single-Institution Experience

**DOI:** 10.7759/cureus.72517

**Published:** 2024-10-28

**Authors:** Pavel Brancik, Vasileios Apostolopoulos, Lubos Nachtnebl, Jakub Rapi, Jakub Liskay, Jan Emmer, Tomas Tomas

**Affiliations:** 1 First Department of Orthopaedic Surgery, St. Anne’s University Hospital and Masaryk University, Brno, CZE

**Keywords:** carpometacarpal joint, cmc joint arthroplasty, dash score, kapandji score, rhizarthrosis

## Abstract

Rhizarthrosis is accompanied by decreased mobility, poor grip, and progressive pain. Surgical treatment, which involves various techniques, is the only definitive solution sparing the joint. First carpometacarpal (CMC) joint arthroplasty helps reduce the discomfort associated with rhizarthrosis and restores joint function.

From 2020 to the end of March 2024, 35 arthroplasties of the first CMC joint have been performed. In this study, the implant survival was recorded at a five-year follow-up. Evaluation of functional outcomes was conducted using preoperative and postoperative Disabilities of the Arm, Shoulder, and Hand (DASH), visual analogue scale (VAS), and Kapandji scores. Two types of implants were used, and their postoperative DASH and Kapandji scores were compared.

The one-year implant survival was 0.97, and the three-year implant survival was 0.86. Range of motion showed significant improvement in patients after the first CMC arthroplasty. The VAS score, which assesses pain, and the DASH score, used to subjectively assess upper extremity disability, showed similar postoperative improvement. There is no significant difference in postoperative outcomes (DASH score and Kapandji score) between the “standard” and “dual mobility” implant types when evaluating postoperative outcomes. One intraoperative and two postoperative complications were observed in this study.

First CMC joint arthroplasty effectively relieves pain, improves range of motion, and enhances functional outcomes in patients with rhizarthrosis. Both implant types demonstrated similar postoperative results in terms of DASH and Kapandji scores. While the study observed a few complications, the overall results support the efficacy of first CMC joint arthroplasty as a reliable treatment option for restoring joint function and reducing pain.

## Introduction

The first carpometacarpal (CMC) joint, also known as the trapeziometacarpal (TM) joint, is a saddle joint with a biconvex shape. The joint is composed of the first metacarpal bone and the trapezium carpal bone [1,2]. The movements in the first CMC joint are flexion, extension (radial abduction), palmar abduction, retroposition, adduction, and opposition [2,3]. The degenerative process of osteoarthritis of the first CMC joint is also called rhizarthrosis and occurs more often in women than in men [4,5]. It can present with pain in the CMC joint; initially, the pain is caused by movement and heavy work, but gradually, the pain may be present at rest and at night. Another symptom of rhizarthrosis is difficulty with movement and weakness of the thumb when grasping huge and weighty objects [4,5]. Because osteoarthritis of the first CMC joint limits the patient's daily activities, rhizarthritis is very immobilizing [5]. On clinical examination, the patient presents with pain in the area of the first CMC joint, limited movement, and a weak grip. During this examination, joint effusion, synovial thickening, and osteophytes are observed [6]. An X-ray is used to confirm the diagnosis of rhizarthrosis. The Eaton-Littler classification describes four stages of CMC arthrosis. It describes the progressive reduction of joint space, the presence of osteophytes, or the development of subchondral changes [5,7].

Besides conservative treatment, definitive treatment includes surgical treatment. There are many surgical techniques, including trapeziectomy with or without ligament reconstruction and interposition, arthroscopy and debridement, arthroplasty, and TM arthrodesis [8]. However, there is no clear consensus on the type of surgical therapy for those patients, and orthopedic surgeons choose the type of treatment based on their experience. The first CMC joint arthroplasty helps to reduce the discomfort associated with rhizarthrosis and restores joint function. The benefits of the first CMC joint arthroplasty include faster recovery and reduced pain. Return to normal activities is significantly faster compared to trapeziectomy. In the advanced stage of rhizarthrosis, the thumb can become deformed, and surgeries, such as arthrodesis and trapeziectomy, cause shortening and poor appearance of the thumb. Only arthroplasty can better correct the deformity and length of the thumb. Complications associated with donor tendon and wrist instability occur with os trapeziumsurgery. These complications do not happen with arthroplasty [9].

Based on the current literature, there remains controversy and limited knowledge about the use and the clinical outcomes of first CMC arthroplasty. The purpose of this study is to evaluate the early functional outcomes and complications of the first CMC arthroplasty at our institution, including a comparison between different implant types.

## Materials and methods

Study design and patient selection

Patients who underwent arthroplasty of the first CMC joint from January 2020 to March 2024 were included in this study. The functional outcomes and complications were assessed. Before surgery, radiographic imaging was conducted to determine the degree of arthrosis of the first CMC joint according to the Eaton-Littler classification. Grade 3 arthrosis was present in 18 cases, and grade 4 arthrosis was present in 17 cases. The Disabilities of the Arm, Shoulder, and Hand (DASH) score was used for the subjective assessment of upper limb disability. Thumb range of motion was also assessed, according to Kapandji. Pain was monitored and assessed using the visual analogue scale (VAS). These scores were compared before and after surgery.

In this study, 35 arthroplasties of the first CMC joint were performed in 31 patients (Table 1). The mean age of the patients who underwent surgery was 58.29 years (±7.21 years). The mean follow-up in this study was 20.7 (±13.16 months) months. Three surgeries were performed on males, and 28 were performed on females. Seventeen surgeries were performed on the right hand, and 18 were performed on the left hand. In 19 cases, the dominant limb was involved, and in 16 cases, the non-dominant limb was involved. The only indication for arthroplasty of the first CMC joint was rhizarthrosis. Patients with posttraumatic arthritis, rheumatoid arthritis, or scaphotrapeziotrapezoid osteoarthritis were not included in this study. The preoperative and postoperative courses were the same for all patients.

**Table 1:** Sample characteristics

Variable	Overall	Standard	Dual mobility	p-values
Number of surgeries	35	14	21	-
Number of patients	31	13	20	-
Age at inclusion (years)	58.29 (±7.21)	57.14 (±6.49)	59.05 (±7.56)	0.25
Follow-up (months)	20.7 (±13.16)	34.5 (±8.26)	11.5 (±5.64)	<0.05
Sex				
Male	3 (9.68 %)	0 (0.00 %)	3 (15.00 %)	0.26
Female	28 (90.32 %)	13 (100.00 %)	17 (85.00 %)	-
Side				
Right	17 (48.57 %)	8 (57.14 %)	9 (42.86 %)	0.31
Left	18 (51.43 %)	6 (42.86 %)	12 (57.14 %)	-
Handedness				
Dominant	19 (54.29 %)	9 (64.29 %)	10 (47.62 %)	0.32
Non-dominant	16 (45.71 %)	5 (35.71 %)	11 (52.38 %)	-

Surgical technique and postoperative care

In all cases, a standardized surgical technique was employed. Patients were positioned supine with their hands on the operating table, and a pneumatic tourniquet was applied to the arm. A dorsal approach was utilized, accessing the joint through the interval between the extensor pollicis brevis and abductor pollicis longus muscles. The joint capsule was incised to expose the joint. The base of the first metacarpal bone was resected. The medullary cavity was prepared and rasped to accommodate the stem of the implant. The surface of the trapezium was resected and rasped accordingly for the cup implantation. A trial cup was inserted into the trapezium, and a trial neck was used to assess stability and range of motion. The joint capsule was reconstructed, and the wound was closed in layers. Postoperatively, a plaster cast was applied to the forearm, and immobilization lasted for three weeks. After three weeks, the cast was removed, and the patient commenced physiotherapy to restore mobility and strengthen the surrounding joint muscles. A gradual reintroduction of full hand loading was initiated three months post-surgery to ensure optimal recovery and function.

Implant characteristic

**Figure 1:**
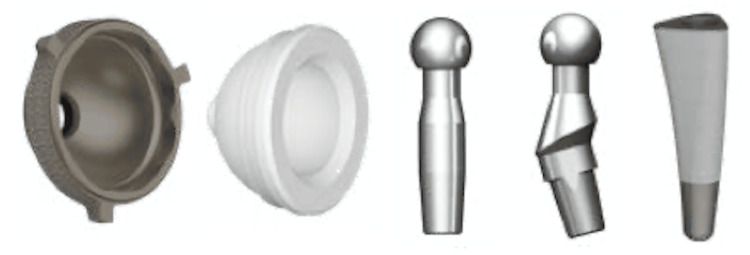
ELiS “standard” endoprosthesis of the first carpometacarpal (CMC) joint used from 2020 to the end of June 2022.

**Figure 2:**
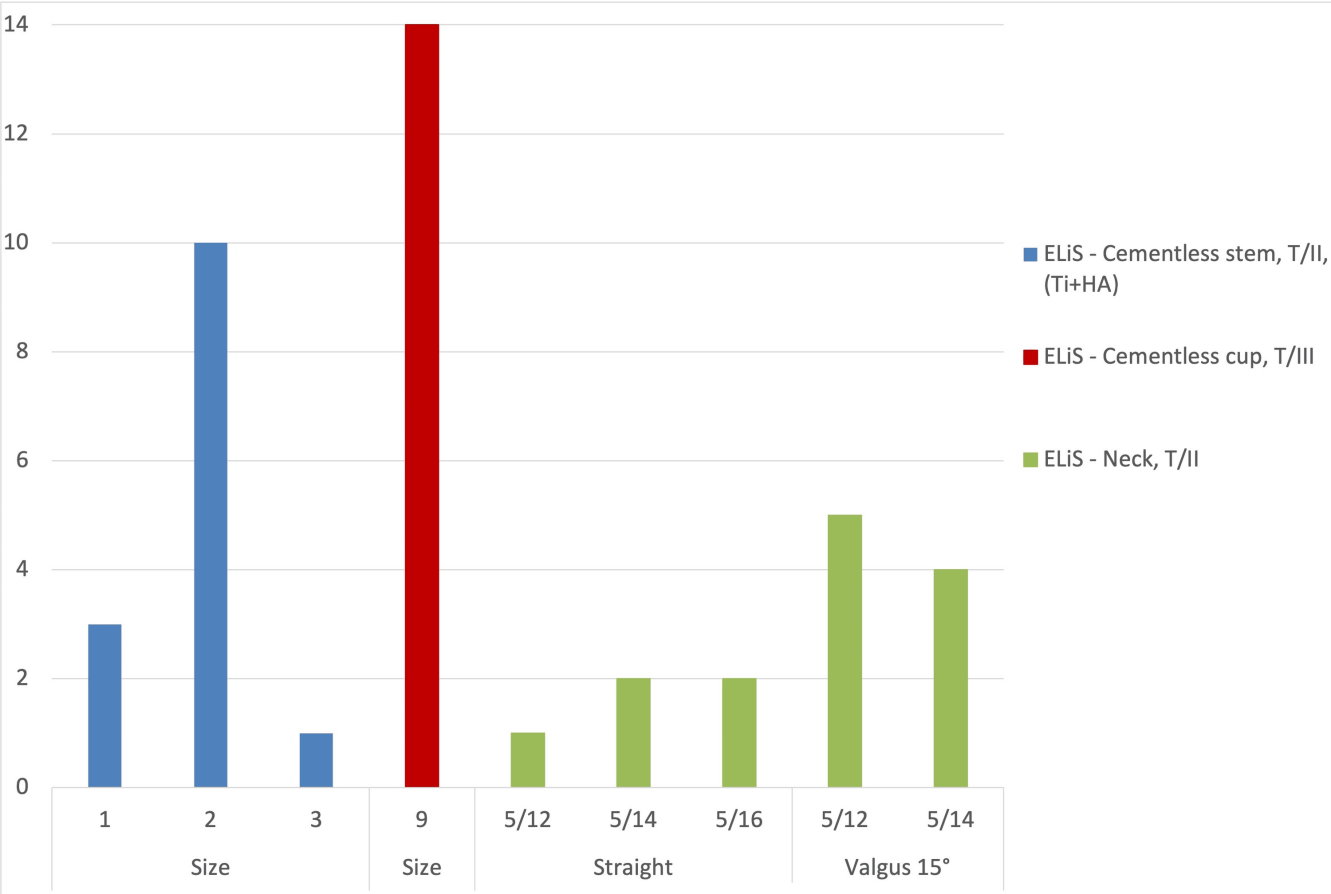
Types and sizes of ELiS “standard” endoprosthesis of the first carpometacarpal (CMC) joint used from 2020 to the end of June 2022.

Implants from Beznoska, s.r.o. (Kladno, Czech Republic), are used at our institution. From 2020 to the end of June 2022, the following stem types were used: the ELiS Cementless Stem, T/II (Ti+HA); the ELiS Cementless Cup, T/III; and the ELiS Neck, T/II. These components are hereinafter referred to as “standard” (Figure 1) [10]. The numbers of implants used above are shown in Figure 2.

**Figure 3:**
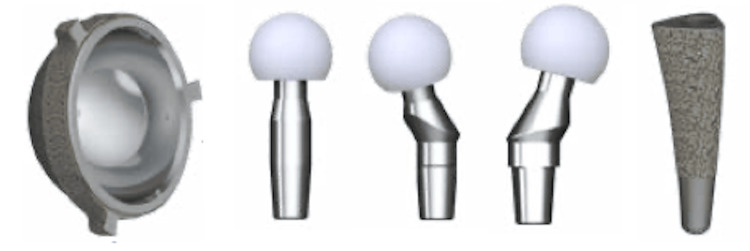
ELiS “dual mobility” endoprosthesis of the first carpometacarpal (CMC) joint used from July 2022 to the end of March 2024.

**Figure 4:**
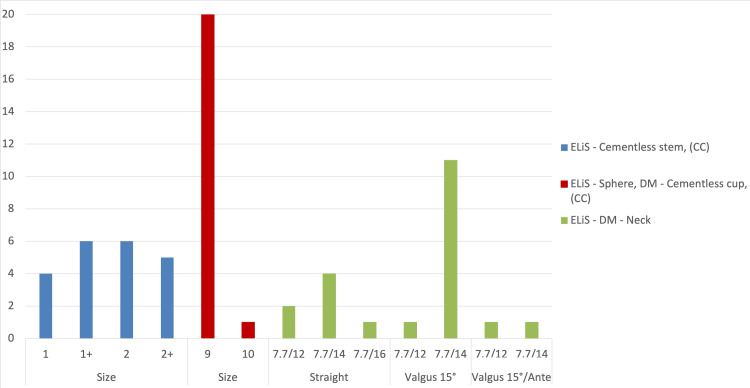
Types and sizes of ELiS “dual mobility” endoprosthesis of the first carpometacarpal (CMC) joint used from July 2022 to the end of March 2024.

In July 2022, there was a transition to the new generation of prostheses. The implantation of stem components with additional intermediate sizes and neck components with dual mobility design was initiated. The following stem types were used: the ELiS Cementless Stem (CC), the ELiS Sphere DM Cementless Cup (CC), and the ELiS DM Neck. These components are hereinafter referred to as “dual mobility” (Figure 3) [10]. The number of implants used above is shown in Figure 4.

Statistical evaluation

Statistical analysis was done using R software (version 4.0.5) (R Foundation for Statistical Computing, Vienna, Austria) in the RStudio (Posit Software, Boston, MA) development environment. Kaplan-Meier analysis was used to evaluate implant survival. Fisher’s exact test was used to evaluate the dependence of two categorical variables. The Mann-Whitney U test was used to evaluate the difference between the functional outcomes of the two groups for a continuous variable. Fisher’s exact probability test was used to compare the proportions between the two groups.

## Results

Implant survival

**Figure 5:**
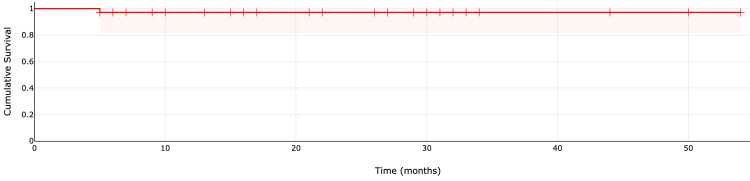
Survival curve of the first carpometacarpal (CMC) joint arthroplasty from 2020 to the end of March 2024.

The implant survival rate was 97% at both one- and three-year follow-ups (Figure 5).

**Figure 6:**
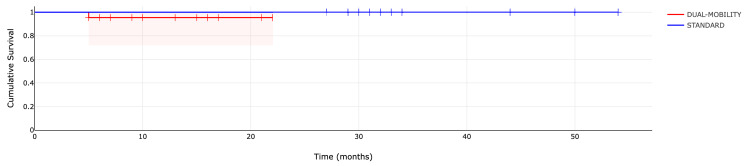
Survival curve of the first carpometacarpal (CMC) joint arthroplasty from 2020 to the end of March 2024: type of implant analysis.

The implant survival rate for the “dual mobility” group was 95% at the 22-month follow-up. In contrast, no implant failures were recorded for the “standard” implant group, with a 100% survival rate at the three-year follow-up (p = 0.41) (Figure 6).

Complications

**Figure 7:**
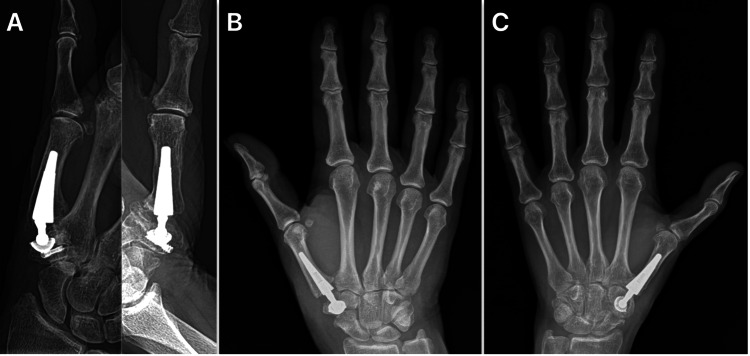
(A) Postoperative radiographic imaging in anterior-to-posterior (AP) and lateral projection of the intraoperative trapezium fracture. (B) Radiographic imaging in AP projection of the postoperative resorption of the trapezium. (C) Radiographic imaging in AP projection of the postoperative fracture of the trapezium following a fall.

During this period, three complications were encountered: one intraoperative and two postoperative. The intraoperative complication occurred during the implantation of the cementless cup. The trapezium was partially split in half during the procedure. To stabilize the trapezium fracture, a compression screw was used (Figure 7A). Postoperatively, the duration of plaster cast fixation was extended to six weeks, and the rehabilitation period was also prolonged. Initially, the patient demonstrated 20° of thumb flexion-extension and 30° of adduction-abduction. However, this range of motion improved over time. At the final follow-up, the patient achieved 40° of flexion-extension and 50° of adduction-abduction, with no associated pain.

The first postoperative complication occurred without any reported trauma. At 5.5 months post-surgery, the patient presented with forearm pain and impaired mobility. A radiographic examination revealed a fracture of the trapezium (Figure 7B). This complication is recent, and a definitive course of treatment has not yet been determined.

The second postoperative complication occurred one year and eight months after the total joint replacement. The patient suffered a fracture of the trapezium axis following a fall (Figure 7C). Given the patient's history of non-compliance and multiple previous falls, arthrodesis was performed to address the issue.

Preoperative vs. postoperative functional outcomes

DASH Score

**Figure 8:**
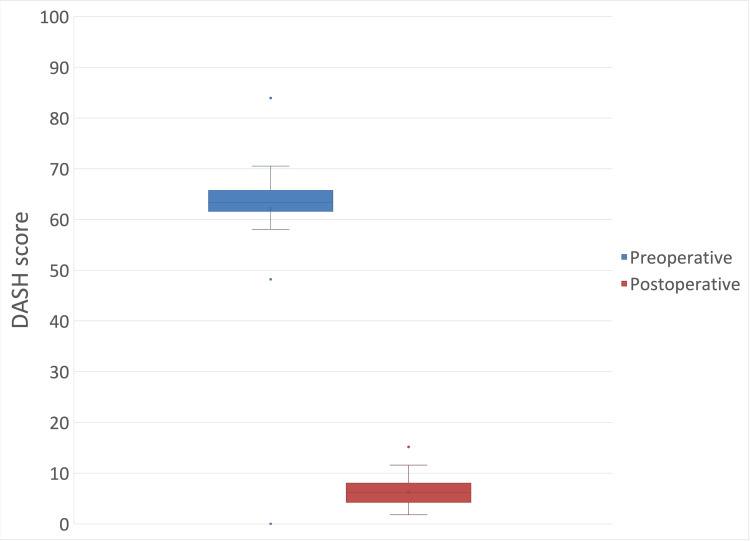
Preoperative and postoperative Disabilities of the Arm, Shoulder, and Hand (DASH) score.

The mean measured preoperative DASH score was 63.96 (± 5.09), and the postoperative DASH score was 6.31 (± 2.87) (mean difference: - 57.65 (95% CI: -59.64 to -55.66); p < 0.05) (Figure 8).

VAS Score

**Figure 9:**
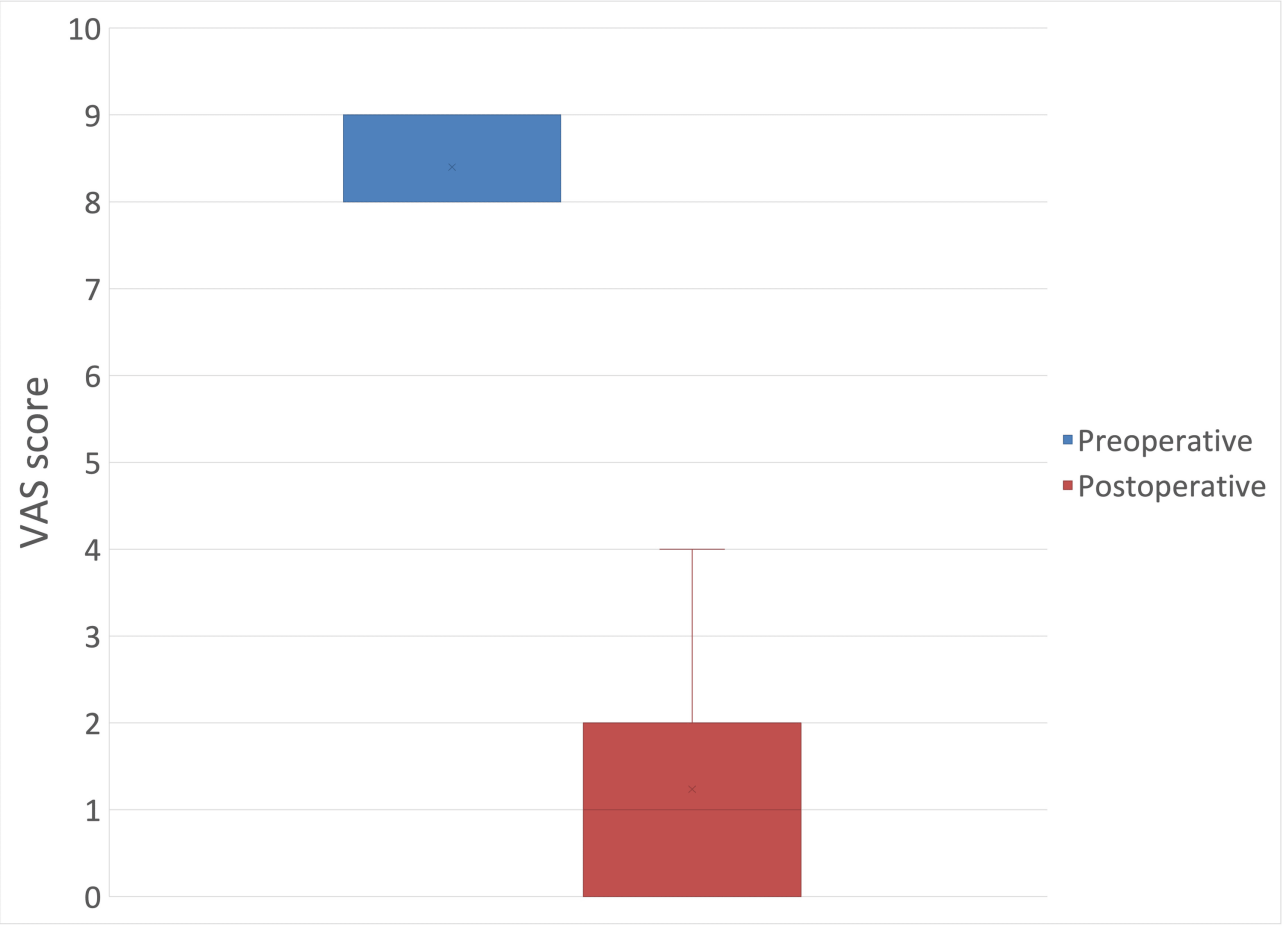
Preoperative and postoperative visual analogue scale (VAS) score.

The mean measured preoperative VAS score was 8.4 (± 0.49), and the postoperative VAS score was 1.24 (± 1.11) (mean difference: -7.16 (95% CI: -7.57 to -6.75); p < 0.05) (Figure 9).

Kapandji Score

**Figure 10:**
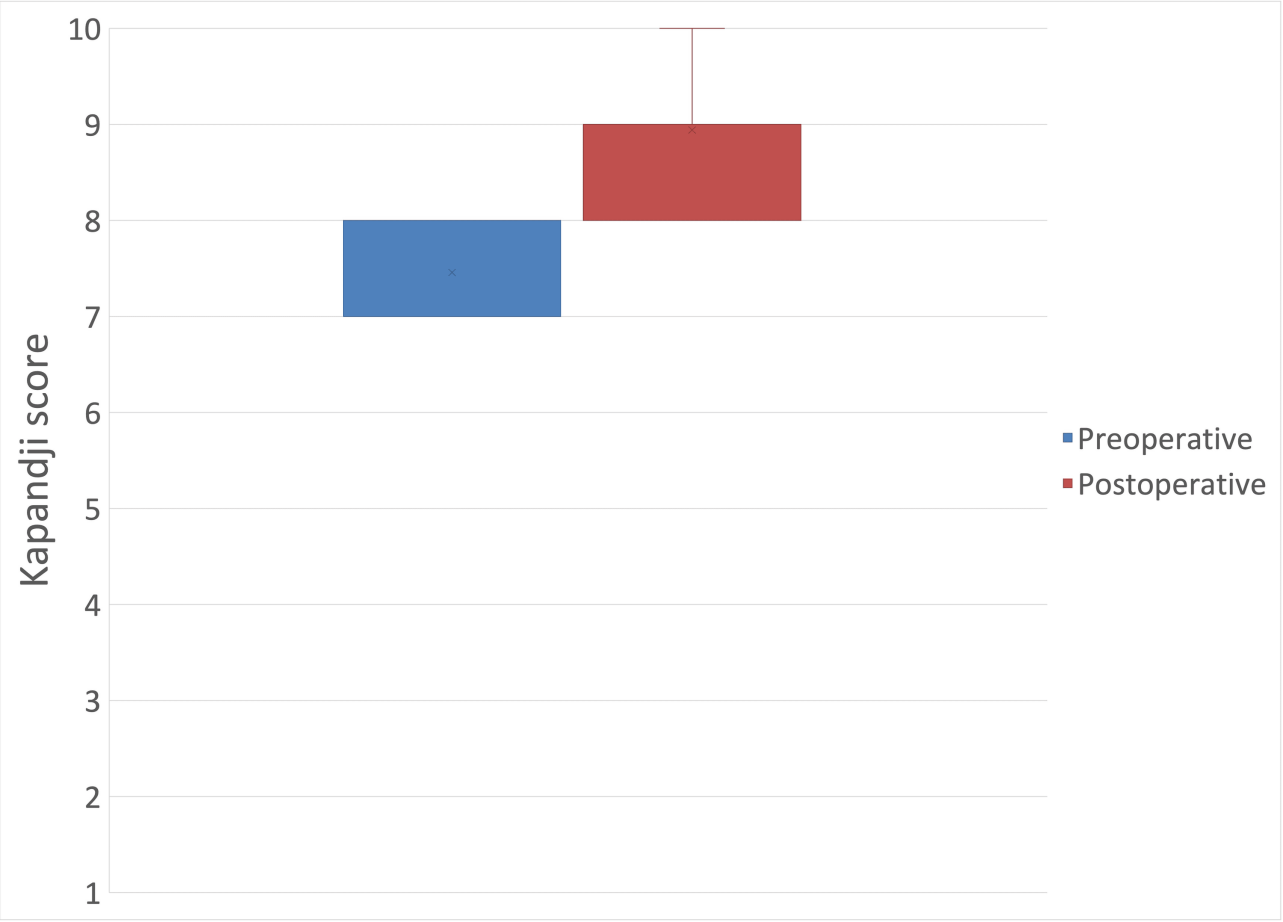
Preoperative and postoperative Kapandji score.

The mean measured preoperative Kapandji score was 7.46 (± 0.5), and the postoperative Kapandji score was 8.94 (± 0.68) (mean difference: 1.48 (95% CI: 1.19 to 1.77); p < 0.05) (Figure 10).

Results by implant type

DASH Score

**Figure 11:**
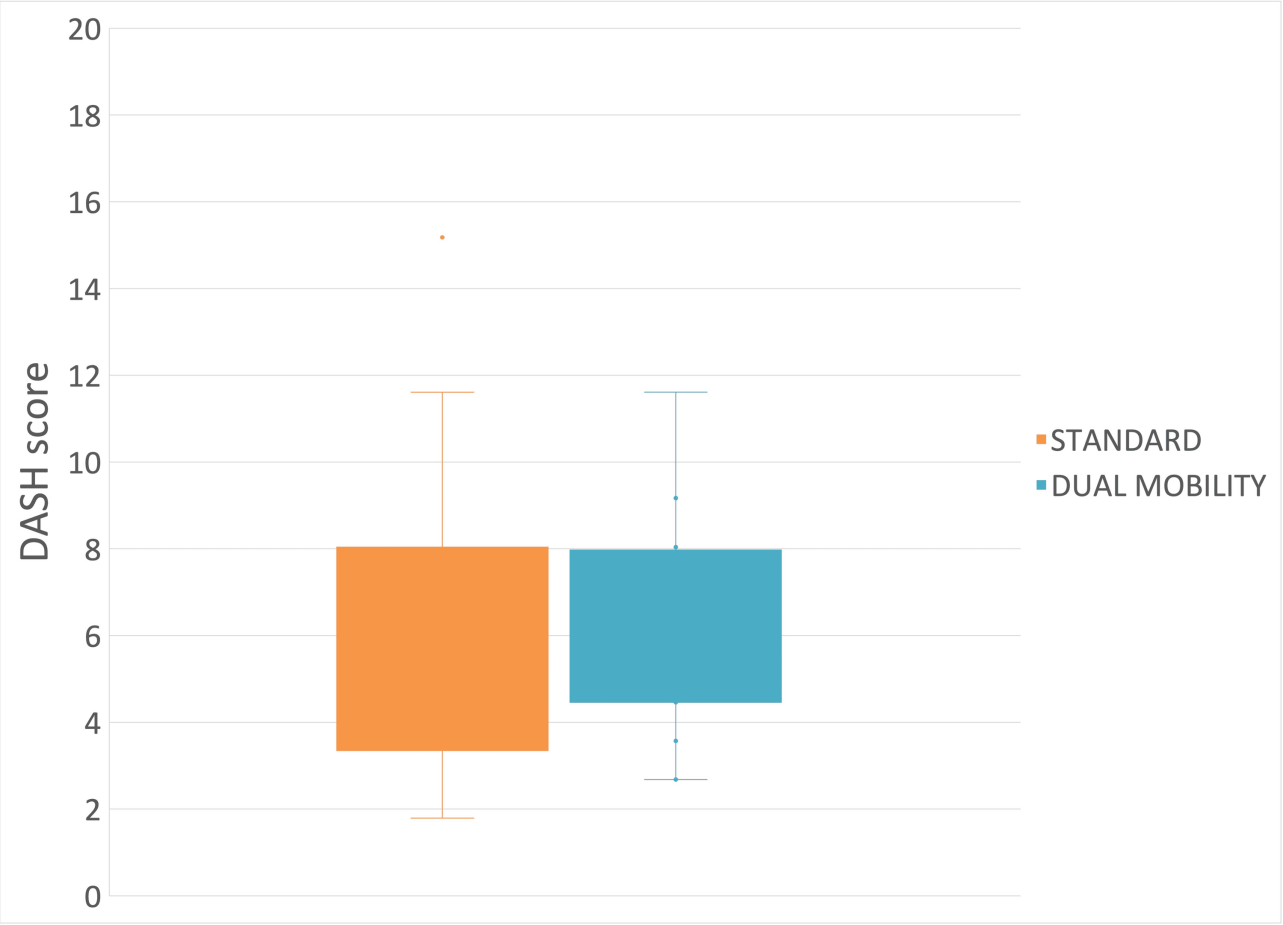
Postoperative Disabilities of the Arm, Shoulder, and Hand (DASH) score between implant types.

The mean measured postoperative DASH score for the “standard” implant type was 6.49 (±3.52), and the postoperative DASH score for the “dual mobility” implant type was 6.19 (±2.31) (mean difference: -0.3 (95% CI: -2.33 to 1.73); p = 0.61) (Figure 11).

Kapandji Score

**Figure 12:**
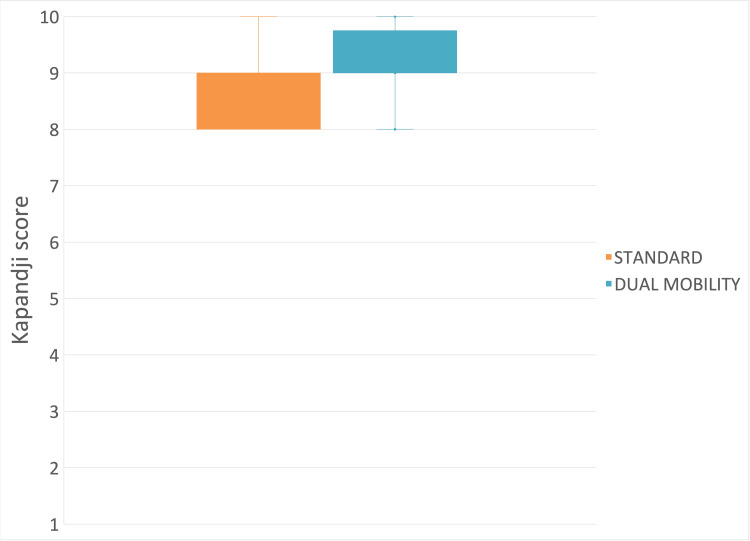
Postoperative Kapandji score between implant types.

The mean measured postoperative Kapandji score for the “standard” implant type was 8.79 (±0.67), and the postoperative Kapandji score for the “dual mobility” implant type was 9.05 (±0.67) (mean difference: 0.26 (95% CI: -0.22 to 0.74); p = 0.33) (Figure 12).

## Discussion

The three-year survival rate of 0.97 is favorable and aligns with previously reported mid-term outcomes for the first CMC joint replacements in existing literature, where survival rates for first CMC joint arthroplasty typically range from 0.80 to 0.90 at the three- to five-year mark. [11-14] However, at the five-year follow-up, the Elektra Single-Mobility Prosthesis (Small Bone Innovations, Morrisville, PA) demonstrated lower implant survival rates. [12] The comparison of survival rates between “dual mobility” and “standard” implants reveals a difference, though it is not statistically significant. Additionally, the disparity in follow-up durations between the two implant groups is noteworthy. 

The intraoperative trapezium fracture, which occurred during implantation of the cementless cup, mirrors challenges described in the literature concerning the technical difficulty of positioning implants in the trapezium [15,16]. Such fractures have been reported in 2-5% of cases involving first CMC joint arthroplasty, and the use of fixation with screws, as in this case, is a common corrective measure [14]. Post-traumatic fractures following CMC arthroplasty are well-documented in cases of patient non-compliance or repeated trauma, particularly in elderly patients or those with osteoporotic bones [17]. Arthrodesis as a salvage procedure for the first complication aligns with standard practices in the literature, where CMC joint fusion is often recommended when revision arthroplasty is not feasible. The spontaneous trapezium fracture without reported trauma is less commonly described but could be related to underlying bone weakness or suboptimal load distribution by the implant [18]. Studies have reported a small percentage of implant-related fractures, particularly with cementless cups, where osteolysis or stress shielding could contribute to late-onset fractures [14,17,18].

The functional outcomes measured by the DASH score showed a significant difference between preoperative and postoperative outcomes. There is a distinct improvement in postoperative outcomes. In their study, Toffoli et al. evaluated preoperative and postoperative Quick Disabilities of the Arm, Shoulder, and Hand (QuickDASH) scores. Their outcomes are consistent with ours, and a significant difference between the two groups was found [19]. Similar outcomes are also found in the study by Lussiez et al. [20]. The Kapandji score revealed a significant increase in the range of motion after surgery. Lussiez et al. also compared preoperative and postoperative Kapandji scores. The outcomes of their study showed the same improvement in movement after surgery as ours [20]. There are other studies evaluating postoperative QuickDASH and Kapandji scores. These values are similar to ours [3,9].

No significant difference in postoperative functional outcomes was found between “standard” and “dual mobility” implants. DASH and Kapandji scores were similar postoperatively between both groups. Frey et al. evaluated the postoperative DASH and Kapandji scores between a single-mobility (Elektra) and a dual-mobility (Moovis (Stryker, Pusignan, France)) prosthesis. In their study, there is also no significant difference between the postoperative DASH and Kapandji scores. Compared to our results, the Kapandji score is similar to ours, but the DASH score is better in our study [12].

There are several limitations to the present study. Firstly, the relatively small sample size of 35 surgeries may limit the generalizability of the findings to a larger population. Additionally, as a retrospective study, the analysis relies on previously collected data, which may introduce inherent biases and limit the ability to control for confounding variables. Another limitation is the relatively short follow-up period, as well as the variability in follow-up durations between the two implant groups, which could affect the comparability of outcomes. Furthermore, the study was conducted at a single institution, meaning it does not account for variations in surgical techniques, post-operative rehabilitation protocols, or patient management practices that might differ across other centers, potentially influencing both functional outcomes and implant survival.

## Conclusions

The findings of the present suggest that first, CMC joint arthroplasty effectively relieves pain, improves range of motion, and enhances functional outcomes in patients with rhizarthrosis. Both implant types (“standard” and “dual mobility”) demonstrated similar postoperative results in terms of DASH and Kapandji scores. The high one-year implant survival rate underscores the early durability of the procedure. While the study observed a few complications, the overall results support the efficacy of first CMC joint arthroplasty as a reliable treatment option for restoring joint function and reducing pain. Longer follow-up and research of the first CMC joint replacement may yield further insights.
